# Transcriptomic Responses of Two Ecologically Divergent Populations of Japanese Mantis Shrimp (*Oratosquilla oratoria*) under Thermal Stress

**DOI:** 10.3390/ani9070399

**Published:** 2019-06-30

**Authors:** Fangrui Lou, Zhiqiang Han, Tianxiang Gao

**Affiliations:** 1 Fishery College, Zhejiang Ocean University, Zhoushan 316022, China; 2 Fishery College, Ocean University of China, Qingdao 266003, China

**Keywords:** crustacea, climate change, intraspecific adaptation difference, muscle functional genomics

## Abstract

**Simple Summary:**

Rising ocean temperature would change the seawater chemistry and affect the external and internal physiology of crustaceans due to their lack of certain efficient temperature regulators. In addition, the infraspecific populations of crustaceans might also have different response strategies to the rising of temperature. Therefore, we identified the transcriptomic variations to the same thermal stress between ecologically divergent populations of *Oratosquilla oratoria*. The aim of this study was to investigate the population-specific function genes and relevant pathways in response to thermal stress in *O. oratoria*. The results showed that gene-expressed variation was in a population-specific pattern, which indicated that the local environment could lead to the evolvement of changes in gene regulation, ultimately leading to adaptive divergences. Additionally, we found several genes with large pleiotropic effects in the Zhoushan population, which might indicate that the regulation mechanisms of the Zhoushan population were more efficient than those of the Qingdao population under same thermal stress. The results provided some novel insights into the local adaptive differences of the infraspecific populations of *O. oratoria* and other crustaceans.

**Abstract:**

Crustaceans are generally considered more sensitive to ocean warming due to their lack of certain efficient regulators. However, the alterations in the physiology and behavior of crustaceans in response to thermal stress differ vastly even among the infraspecific populations of heterogeneous landscapes. Consequently, understanding the impact of temperature fluctuation on crustacean infraspecific populations might be essential for maintaining a sustainable persistence of populations at existing locations. In the present study, we chose the Japanese mantis shrimp (*Oratosquilla oratoria*) as the representative crustacean population, and conducted transcriptome analyses in two divergent *O. oratoria* populations (the Zhoushan and Qingdao populations) under same thermal stress (20–28 °C) to identify the population-specific expression response to thermal stress. The results showed significant differences in gene expressions, GO terms and metabolic pathways between the two populations. We hypothesized that intraspecific mutations in the same or different genes might lead to thermal adaptive divergences. Temperature increases from 20–28 °C produced significant enrichment in GO terms and altered the metabolic pathways in the Zhoushan population despite the lack of differentially expressed unigenes. Therefore, several functional genes with large pleiotropic effects may underlie the response to thermal stress in the Zhoushan population. Furthermore, the most significantly enriched biological processes of the Qingdao population were associated with the state or activity of cells and its significant enriched pathways with genetic information processing as well as immune and environmental information processing. In contrast, the differentially regulated unigenes of the Zhoushan population were primarily involved in the regulatory cellular and transcription processes and the most significant pathways found were metabolic and digestive. Consequently, the regulatory mechanisms of the Zhoushan population are probably more efficient than those of the Qingdao population under the same thermal stress.

## 1. Introduction

The current global warming trend is thought to cause widespread effects on the structure and functioning of global fauna and ecosystem processes. Although the ocean has a higher heat capacity and its temperature is relatively stable in comparison with the land, the world’s oceans are also continuously warming and the average ocean temperature is predicted to increase approximately 3 °C by the end of the century [1]. It is likely that the rising ocean temperature will change the seawater chemistry and then affect the external and internal physiology of marine organisms [2]. Sunday et al. also considered that most marine organisms belong to poikilotherms and therefore are particularly vulnerable to a rise in temperature of their surroundings [3]. Thus, a rapid regulation capacity and a higher physiological endurance to rising temperatures are very necessary because they can help organisms reduce maladaptive responses [4]. However, responses to rising temperatures might vary in different species. For instance, evidence has suggested that microhabitat species might encounter more pressure than macrohabitat species when habitat temperature continues to rise [5]. In addition, tropical species are expected to be particularly sensitive to increases in temperature because relatively stable thermal environments play an important role in their ecological evolutionary processes [6]. However, the infraspecific populations of heterogeneous landscapes might also have different response strategies to rising temperatures. This may be due to their physiological characteristics and biological interactions being directly affected by their local habitat environment and ultimately leading to genetic adaptation differences [7,8]. Consequently, as oceans become warmer, we can expect to see different pressure on the infraspecific populations that live in different environments. Elucidation of these variations at the transcriptome level would facilitate our understanding of whether these infraspecific organisms have the same ability to cope with ocean warming.

Although numerous studies have revealed that differential habitat temperatures may lead to evolutionarily significant units (ESUs) of many crustacean species [9,10], these studies were constrained by neutral markers that became an impediment for the precise evaluation of the infraspecific adaptability divergence of crustaceans. Recently-developed next-generation sequencing technology has provided a convenient and highly effective solution for biological study. As a result, several studies have more accurately estimated the genetic divergence based on reduced-representation sequencing [11,12], but they could not explain how the temperature fluctuations affected the regulatory mechanisms of marine organisms. However, RNA sequencing (RNA-seq) has clear advantages over the aforementioned approaches and can reveal the complex dynamics of the regulatory processes with both accuracy and sensitivity. To date, the RNA-seq approaches have been successfully used to analyze the regulatory mechanisms of interspecific organisms [13,14,15], and some functional genes and physiological pathways were also identified [16,17,18]. Therefore, with respect to infraspecific organisms in their heterogeneous environments, RNA-seq can also serve as a useful technique for identifying the genetic divergences associated with local environments, which can then be subjected to a further examination of the thermal adaptability of infraspecific organisms [19,20].

**Figure 1:**
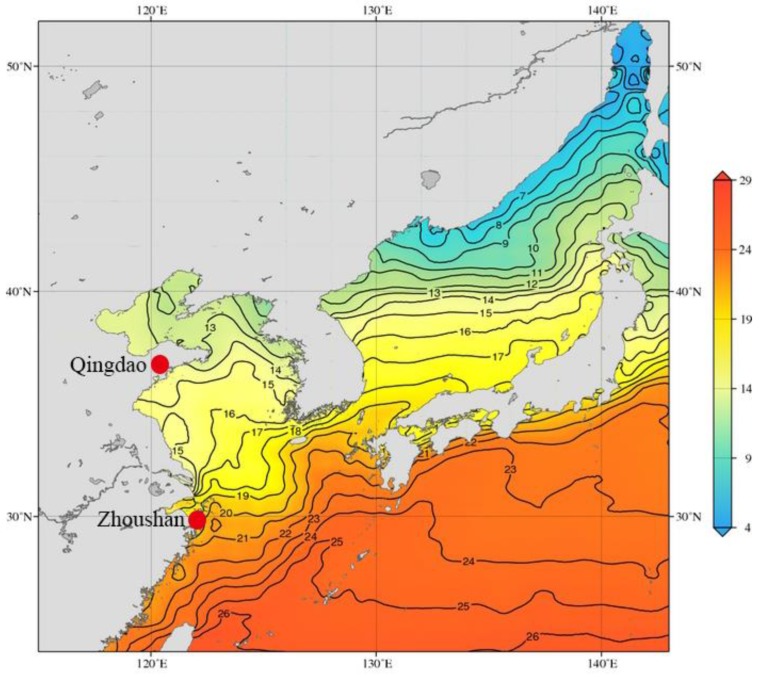
Map of East Asia showing annual surface temperature (°C; 10-degree grid), based on Johnson and Boyer (2015) [24].

It is still worth noting that most marine calcifying organisms (e.g., crustaceans) are generally considered more sensitive to ocean warming because they lack certain efficient regulators [21]. Thus, it is necessary to discuss the long term ability of the crustacean infraspecific organisms to respond to ocean warming. As a crustacean representative, *Oratosquilla oratoria* (De Haan, 1844) spans a wide geographical area throughout the tropical, subtropical and temperate coastal waters, and its suitable survival temperature ranges from 20–27 °C [22]. Across this wide distribution, the *O. oratoria* might encounter highly heterogeneous environmental elements. In fact, Du et al. divided two *O. oratoria* geographical populations (the Yellow Sea and East China Sea populations) [23] and suspected that their genetic differentiation might be associated with temperature variability (Figure 1; [24]). Furthermore, since their habitat differences might ultimately lead to genetic divergence, it is not surprising that two *O. oratoria* populations vary in thermotolerance. Therefore, *O. oratoria* provided an ideal research source for investigating the thermal response variation of crustacean infraspecific populations.

The muscle is the largest energy and amino acid pool in the maintenance of homeostasis in ocean animals and because it exhibits a high surface area directly suspended in the water. Therefore, the muscle might be associated with thermal response. However, whether the *O. oratoria* infraspecific organisms have the same ability to cope with thermal stress is not well understood. In the present study, we determine the transcriptome changes that occur in response to thermal stress in the muscle of two divergent *O. oratoria* populations. Specifically, in order to avoid population mixture, the mitochondrial DNA *Cytochrome oxidase subunit I* (*COI*) and the *control region* sequences were used to distinguish between the *O. oratoria* populations. Then, 20 °C and 28 °C were selected as the control and heat stress treatment temperatures, respectively. The aim of this research was to identify the population-specific function genes and relevant pathways in response to thermal stress in *O. oratoria*. Furthermore, these results can further enhance our understanding of how environmental heterogeneity and climate change have contributed to adaptive diversification within lineages.

## 2. Materials and Methods

### 2.1. Ethics Approval and Participation Consent

*O. oratoria* is not an endangered or protected species in China nor in other countries. In addition, all *O. oratoria* collection and anatomy experiments were conducted in accordance with the ‘Guidelines for Experimental Animals’ of the Ministry of Science and Technology (Beijing, China; No. [2006] 398, 30 September 2006). It should be noted that frost anesthesia was made to minimise the suffering of all animals.

### 2.2. O. oratoria Maintenance and Heat Exposure

The *O. oratoria* were collected from the coastal waters of Qingdao and Zhoushan regions in China, which belong to the Yellow Sea and the East China Sea, respectively (Figure 1). All *O. oratoria* were dispatched into separated aquariums with recirculating, aerated seawater (28 salinity) and acclimated over a 24 h period at 20 °C. The heating schemes of the two populations were similar, involving water temperature graded increases from 20 °C to 28 °C, slowly, at a rate of 1 °C per day and acclimated over a 24 h period at 28 °C. Each population was divided into a control group (20 °C) and a treatment group (28 °C). Muscle tissues for transcriptome analyses were collected in four testing groups: Qingdao control group (QD_control_), Qingdao treatment group (QD_treatment_), Zhoushan control group (ZS_control_), and Zhoushan treatment group (ZS_treatment_). Three adult females per testing group were euthanised and the muscles were then extracted using sterilized scissors and forceps [25]. Subsequently, 12 individual muscles (2 populations × 2 temperature stages × 3 biological replicates) were separately snap-frozen in liquid nitrogen and stored at −80 °C prior to the following experiments. 

Mitochondrial DNA *COI* (LCO1490: 5′-GGTCAACAAATCATAAAGATATTGG-3′; HCO2198: 5′-TAAACTTCAGGGTGACCAAAAAATCA-3′) and the *control region* sequences (OO-F1: 5′-TCAAATAGAAAACAAATAGCCAG-3′; OO-F2: 5′-CATAATTTATCCTATCAAGATAATC-3′) were amplified and used to distinguish between the two populations of *O. oratoria* [26,27]. 

### 2.3. Total RNA Extraction and Illumina Sequencing

Total RNA of individual muscles was extracted using a standard Trizol Reagent Kit (Huayueyang Biotech Co. Ltd., Beijing, China), in accordance with the manufacturer’s protocol, and quantified using Agilent 2100 Bioanalyzer (Agilent Technologies, Santa Clara, CA, USA). We then purified the mRNA from the total RNA (4 μg) using the RNA Purification Beads (Illumina, San Diego, CA, USA). The remaining RNA was cleaned three times using the Beads Binding Buffer (Illumina, San Diego, CA, USA) and the eluted RNA were incubated at 94 °C for 8 min. Fragmentation buffer (Illumina, San Diego, CA, USA) was applied to lyse the mRNA into fragments of a suitable size and the fragmented mRNA was used to construct a cDNA library using TruSeq Stranded mRNA LT Sample Prep Kit (Illumina, San Diego, CA, USA). Afterward, A-Tailing Control (Illumina, San Diego, CA, USA) and Ligation Control (Illumina, San Diego, CA, USA) were applied to A-tailing and adapter ligation of the double stranded cDNA, respectively. Then, the cDNA libraries were diluted to 10 pM and quantified using Agilent 2100 Bioanalyzer (Agilent Technologies, Santa Clara, CA, USA). The libraries were sequenced on the Illumina HiSeq 2000 (Illumina, San Diego, CA, USA) across one lane with paired-end 150 bp.

### 2.4. De Novo Assembly, Gene Expression Variation, and Gene Annotation

All raw reads in the FASTQ format were filtered by removing the reads with sequencing adaptors, unknown nucleotides (N ratio > 10%) and low quality (quality scores ≤ 5). We then used the Trinity package (version 2.0.6; [28]) on the de novo assembled all remaining high-quality reads from 12 samples and the redundancy sequences were removed using the Tgicl (Linux x86) software package, additionally splicing the longest unigenes (universal genes) for further analyses. In order to analyze the gene expression variation, we mapped clean reads of individual sample to a multi-fasta file of all unigenes using BWA-mem [29] and the expression level of the unigenes overall was normalised to determine the FPKM (Fragments per kilobase of exon model per million mapped fragments) using RSEM and Bowtie2 at default settings [30]. The number of differentially expressed unigenes between the two populations was quantified by generating Venn diagrams. Furthermore, we performed homology searches to analyze the functional classifications by comparing all unigenes against the NR, NT, Swiss-Prot, KEGG, COG, and GO databases using the Blastx alignment (E-value < 0.00001).

### 2.5. Testing for Population-Specific Genetic Differentiation

We hypothesized that intraspecific mutations in the same or different genes might lead to local adaptive divergences. To test this hypothesis, we first included a population as an independent variable and assessed whether there was genetic differentiation between the two populations. To quantify the differential gene expressions between populations, we treated the FPKM of Qingdao population as a control value and conducted pairwise comparisons with the FPKM of Zhoushan population inhabiting same temperatures (QD_control_-vs-ZS_control_ and QD_treatment_-vs-ZS_treatment_) using edgeR package with the following parameters: FDR ≤ 0.01 and |log_2_FC| ≥ 2. Furthermore, we assessed which physiological functions might cause genetic differentiation, ultimately leading to adaptability divergence in different *O. oratoria* populations. To do so, we first calculated and compared the enriched functional categories of each differentially expressed unigene between two experimental pairs. Then, pathway enrichment analyses for each population were performed based on the KEGG database [31]. Finally, we constructed pictures using R software to visualize the variations between the two populations.

### 2.6. Identifying Expression Responses to Thermal Stress

To test for local adaptation, expression responses to thermal stress were identified separately for each population. We included temperature factors as independent variables and then differentially expressed unigenes of two experiment pairs (QO_control_-vs-QD_treatment_ and ZS_control_-vs-ZS_treatment_) were identified using edgeR package (http://bioconductor.org), and FDR ≤ 0.01 and |log_2_FC| ≥ 2 were used as the filtering thresholds. Venn diagram was applied to quantitatively analyze the number of differentially regulated unigenes of two experiment pairs. Furthermore, we assessed the biological functions of each population to explore the potential functional consequences associated with local temperature adaptation. GO term and pathway enrichment analyses were conducted to evaluate the differential evolutionary adaptation between the two populations. We also constructed pictures using R software (https://CRAN.R-project.org/) to visualize the differential expression responses between them.

### 2.7. Quantitative Reverse Transcription PCR (qRT-PCR) Validation

qRT-PCR was applied to validate the transcriptomic data. Within each of the categories for up- and down-regulated unigenes, five randomly selected unigenes or contigs were applied to the qRT-PCR analysis and the gene-specific primers were designed using the Primer Premier 5.0 (Table 1). In addition, the *β-actin* (Forward, 5′-ATCGTTCGTGACATTAAGGA-3′; Reverse, 5′-CAAGGAATGAAGGCTGGAA-3′) and 18S rRNA (Forward, 5′-GAAGGATTGACAGATTGAGAG-3′; Reverse, 5′-GTAGCGACGGACACATAT-3′) were chosen as reference genes for internal standardization. Furthermore, standard curves were constructed to identify the ideal dilution times of the cDNA samples and were used as calibrators. A total of 12 cDNA samples were diluted 20-fold using nuclease-free water and were used as templates for PCR. Furthermore, the qRT-PCR analysis was designed following the manufacturer’s instructions for the SYBR® Premix Ex TaqTM (Tli RNaseH Plus) RR420A (TaKaRa Biotech Co., Ltd., Dalian, China). A reaction system of 25 μL was amplified using the ABI PRISM 7300 Real-Time PCR System (Applied Biosystems, Thermofisher Scientific, MA, USA). Three parallel experiments for every cDNA template were performed to increase the veracity of the result. After the PCR program, the data were analyzed with ABI7300 SDS software (Applied Biosystems, Thermofisher Scientific, MA, USA). The relative expression levels of all target unigenes, or contigs, were calculated by the log22-ΔΔCT analysis method (ΔCT = CT_target unigene_ − CT_reference gene_, ΔΔCT = ΔCT_treatment_ − ΔCT_control_).

## 3. Results

### 3.1. Phylogenetic Relationship, Illumina Sequencing, and Annotation of the O. oratoria Muscle Transcriptome

**Figure 2:**
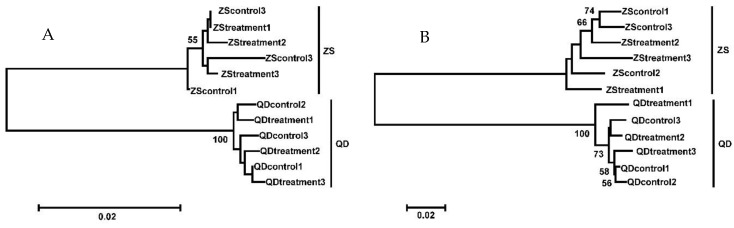
Neighbor-Joining (NJ) tree of the *Cytochrome oxidase subunit I* (*COI*) (**A**) and the *control region* (**B**) sequences constructed using the Kimura 2P distance for *O. oratoria*. QD: Qingdao population; ZS: Zhoushan population.

Mitochondrial DNA *COI* and *control region* sequences were used to identify the phylogenetic relationship of *O. oratoria* in this experiment. The sequencing results of the *COI* and *control region* were shown in Supplementary Files S1 and S2, respectively. The Neighbor-Joining (NJ) trees were constructed using the complete data set of 12 individuals. Results identified that the Qingdao and Zhoushan samples, in the present study, belonged to two distinct lineages (Figure 2).

We obtained the sequencing information of 12 individuals in the present study and these details are listed in Table 2. The statistics for the de novo assembly showed that the number of merged transcriptomes was 130,102,343 bp, with an N50 of 2760 bp, and all unigenes were composed of 30,539 distinct clusters and 56,816 distinct singletons. The transcriptomic raw reads in this publication are archived on the NCBI Short Read Archive (SRA, SRR7346280; SRR7346279; SRR7346288; SRR7346287; SRR7346282; SRR7346281; SRR7346284; SRR7346283; SRR7346286; SRR7346285; SRR7346278; and SRR7346277) under BioProject PRJNA475657. The assembled and annotated transcript has been deposited at the DDBJ/EMBL/GenBank under the accession number GGQQ00000000. The version described in this paper represents the first version, GGQQ01000000. Furthermore, a total of 60,013 unigenes were annotated based on protein databases using the Blastx alignment. Of all annotated unigenes, 50,668, 39,785, 39,340, 37,790, 22,840, and 13,041 unigenes had significant matched with the sequences in the non-redundant protein sequences (NR), nucleotide sequences (NT), swiss prot protein sequence (Swiss-Prot), kyoto encyclopedia of genes and genomes (KEGG), clusters of orthologous groups of proteins (COG) and gene ontology (GO) databases, respectively.

### 3.2. Quantifying Gene Expression Variation

**Figure 3:**
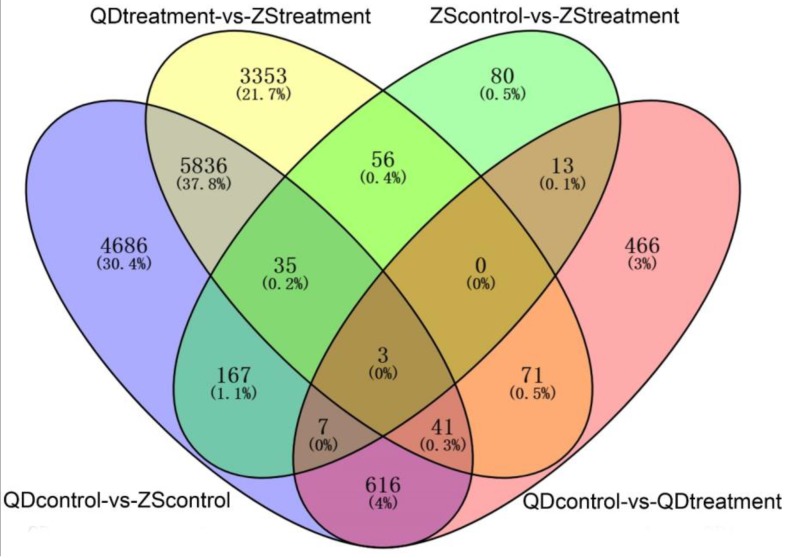
Global overview of the differentially expressed unigenes based on a Venn diagram (ZS_control_: Zhoushan population at 20 °C; ZS_treatment_: Zhoushan population at 28 °C; QD_control_: Qingdao population at 20 °C; QD_treatment_: Qingdao population at 28 °C).

In the present study, the differentially expressed unigenes among four experiment pairs (QD_control_-vs-ZS_control_, QD_treatment_-vs-ZS_treatment_, QD_control_-vs-QD_treatment_, and ZS_control_-vs-ZS_treatment_) were identified by setting the criterions of |Log2FC| ≥ 2 and FDR < 0.05. A Venn diagram was applied to quantitatively reveal the number of differentially expressed unigenes (Figure 3). The results showed that a substantial overlap of differentially expressed unigenes was observed in different pairs, although just three unigenes (CL15652.Contig1_All, Unigene38717_All, Unigene5742_All) were significantly differentially expressed. According to the annotation information, CL15652.Contig1_All, Unigene38717_All, Unigene5742_All may be the homologous sequence of *Craniofacial development protein 2*, *C-type lectin 2* and *beta-lactamase hcpA*.

### 3.3. Population-Specific Gene Expression

**Figure 4:**
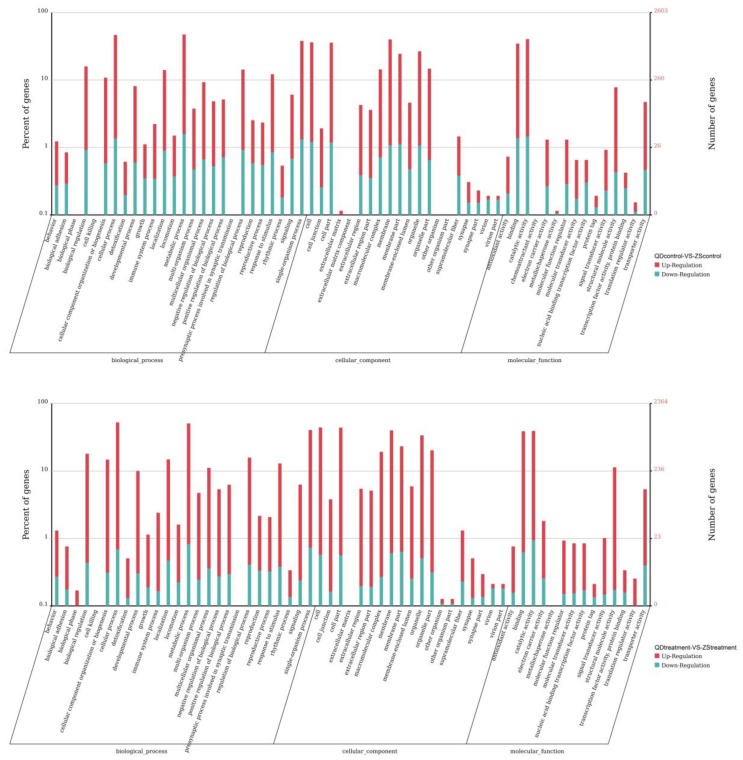
Results of the enrichment analysis of Gene Ontology (GO) terms of the differentially expressed unigenes between two populations (ZS_control_: Zhoushan population at 20 °C; ZS_treatment_: Zhoushan population at 28 °C; QD_control_: Qingdao population at 20 °C; QD_treatment_: Qingdao population at 28 °C).

In the present study, we compared the gene expression between populations. The results indicated that the number of differentially expressed unigenes between the two populations studied were highly significant and that this number decreased with the degree of thermal stress increase (11,391 at 20 °C and 9395 at 28 °C). Furthermore, in comparison with the Qingdao population, a total of 6477 (56.86%) and 6188 (65.86%) upregulated unigenes were obtained from the Zhoushan population at 20 °C and 28 °C, respectively. As predicted, we also found that significant differences existed in the biological processes between the two populations (Figure 4). At 20 °C, only oxidoreductase activity (GO:0016491) and acyl-CoA dehydrogenase activity (GO:0003995) were significantly enriched in the functional responses of the two populations (Table 3). In contrast, the amounts of GO terms were much higher at 28 °C, with about 194 significantly enriched terms that appeared in the comparison of the two populations. For brevity, we only discuss the top 10 enriched terms (Table 3). 

**Figure 5:**
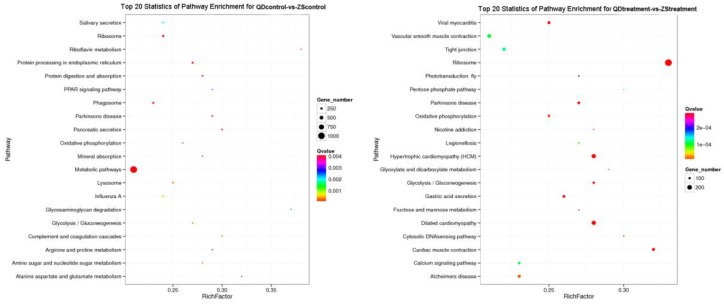
Top 20 pathway enrichment statistics for the two populations at different temperatures (ZS_control_: Zhoushan population at 20 °C; ZS_treatment_: Zhoushan population at 28 °C; QD_control_: Qingdao population at 20 °C; QD_treatment_: Qingdao population at 28 °C).

We recorded the networks of molecular interactions in the cells and the variants specific to particular organisms by comparing the differentially expressed unigenes to the KEGG pathway. At 20 °C, a total of 40 pathways were significantly enriched in the KEGG database (Q ≤ 0.05) and the top 20 statistically significant KEGG (https://www.kegg.jp/) classifications are shown in Figure 5. The results showed some of the digestive- and metabolism-related pathways which were predicted in the KEGG database (ko04972, ko01100, and associated pathways). However, the population-specific expression responses and molecular interactions were lower at 28 °C, with 27 differentially regulated pathways found to be significantly enriched in the two populations (see Figure 5 for the top 20 pathway enrichment statistics). These included the pathways associated with genetic information processing and immunity (ko03010, ko05410, and other associated pathways). For brevity, we only discuss the top 10 statistically significant KEGG classifications of the two populations.

### 3.4. Differentially Expressed Responses of the Two Populations Exposed to the Same Thermal Scheme

To elucidate the pattern of gene expression under thermal stress, we first compared the number of differentially expressed unigenes of each population. The results indicated that the gene expressed differences were closely related to the temperature variation. When the temperature increased from 20 °C to 28 °C, a total of 361 and 1217 differentially expressed unigenes were obtained from the Zhoushan and Qingdao populations, respectively. However, 69.23% (243 up- and 118 down-expressed) and 20.14% (246 up- and 971 down-expressed) up-expressed unigenes were obtained from the Zhoushan and Qingdao populations, respectively. Therefore, we must take into account both the number and the degree of differentially expressed unigenes when conducting transcriptome research. 

**Figure 6:**
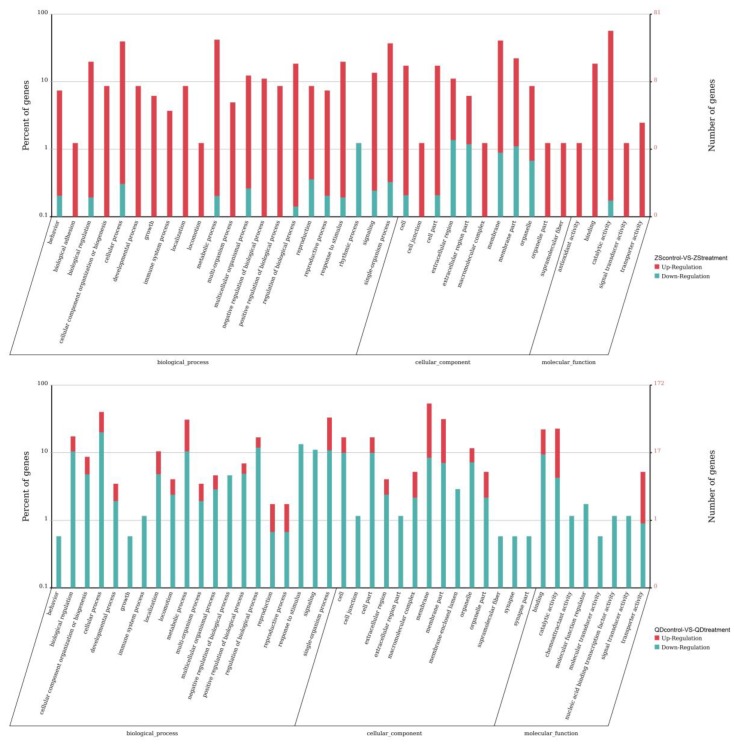
Results of the enrichment analysis of GO terms of differentially expressed unigenes of each population exposed to thermostress (ZS_control_: Zhoushan population at 20 °C; ZS_treatment_: Zhoushan population at 28 °C; QD_control_: Qingdao population at 20 °C; QD_treatment_: Qingdao population at 28 °C).

To identify the functional changes potentially associated with temperature adaptation of the two populations, we conducted a GO term enrichment analysis for differentially expressed unigenes of each population when exposed to the same thermal stress. The results showed that 221 GO terms were enriched in the Zhoushan population and about 26.24% of the terms were statistically significant (Figure 6). For brevity, we only discuss the top 10 enriched terms (Table 4). While the instances of the GO terms were much higher (504 terms), there were only 19 GO terms that were significantly enriched in the Qingdao population (Figure 6; see Table 4 for the top 10 enriched terms). Differentially expressed unigenes in the Qingdao population were predominately associated with the state or activity of cells. These included the processes of cellular movement, cellular secretion, enzyme production, gene expression and cellular apoptosis (GO: 0031435, GO: 0030968, GO: 0034620, GO: 0035967 and other associated terms).

**Figure 7:**
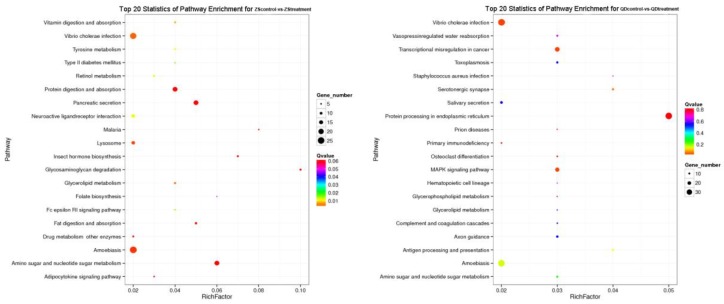
Top 20 pathway enrichment statistics for each population exposed to the same thermal stress (ZS_control_: Zhoushan population at 20 °C; ZS_treatment_: Zhoushan population at 28 °C; QD_control_: Qingdao population at 20 °C; QD_treatment_: Qingdao population at 28 °C).

The differentially expressed unigenes in each pair (ZS_control_-VS-ZS_treatment_, QD_control_-VS-OQ_treatment_) were then used to identify the enriched KEGG pathways. There were 154 and 174 pathways found to be differentially regulated between the Zhoushan and Qingdao populations exposed to the same thermal stress. However, the results also indicated that 18 and 4 pathways were significantly enriched in the Zhoushan and Qingdao populations, respectively (Q ≤ 0.05; see Figure 7 for the top 20 pathway enrichment statistics). Differentially expressed unigenes in the Zhoushan population were predominately associated with the pathways of the metabolism (ko00520 and ko00531) and the digestive system (ko04972 and ko04974). In contrast, the differentially expressed unigenes in the Qingdao population were enriched for pathways associated with genetic information processing (ko04141), immune (ko05202 and ko05110) and environmental information processing (ko04010).

### 3.5. Validation of the Transcriptome Data by qRT-PCR

**Figure 8:**
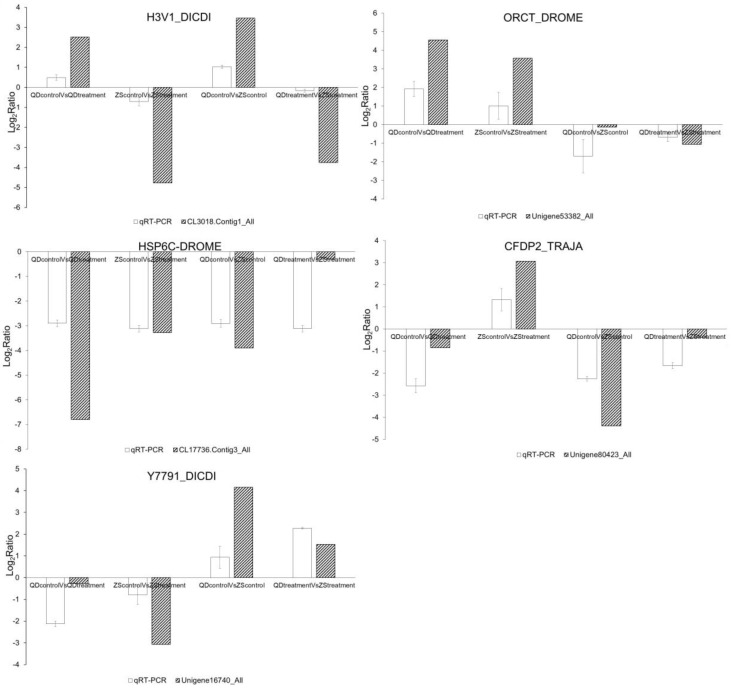
The relative change of the transcriptome data and the qRT-PCR data of each pair (ZS_control_: Zhoushan population at 20 °C; ZSt_reatment_: Zhoushan population at 28 °C; QD_control_: Qingdao population at 20 °C; QD_treatment_: Qingdao population at 28 °C).

We selected five target unigenes, or contigs, to evaluate the transcriptome data of each experimental pair based on the qRT-PCR analysis. For these candidate unigenes, or contigs, the variation trend in expression was concordant between the qRT-PCR data and the transcriptome data, although the values derived from both analytical methods did not perfectly match (Figure 8). Consequently, the results indicated that the transcriptome data were credible.

## 4. Discussion

Crustaceans are under increasing pressure due to elevated water temperatures [21]. Their ability to cope with ocean warming is critical for our understanding of the probability of crustacean sustainability [32]. Nevertheless, there are variations in the rate of and extent to which different populations of crustaceans respond to ocean warming as a result of their long-term habitat differences [7]. As a crustacean representative, *O. oratoria* (De Haan, 1844) spans a wide geographical area and thus different habitats in which temperatures might affect the competition and resource predation regimes and the distribution of the *O. oratoria* populations [22]. Transcriptome sequencing has been widely employed as an effective and accessible approach for understanding many fundamental evolutionary questions when no genome sequencing data are available [33,34,35]. Consequently, this study examined the thermal response differences of two *O. oratoria* populations to better investigate the infraspecific temperature adaption differences in *O. oratoria*. Furthermore, the present study also provided a foundation from which to further our understanding of how different crustacean populations adapt to ocean warming. 

It is undeniable that although there is a conservative transcriptome response in all tissues of the organism under thermal stress, actually each tissue will show highly different responses that are associated with their physiological functions. Muscle tissue is the largest energy and amino acid pool in the maintenance of homeostasis, and it best demonstrates the effect of temperature stress on marine organisms. Additionally, the muscle may exhibit remodeling in response to temperature stress, thereby ensuring that the organism adapts to locomotory activity and load. Therefore, muscle tissues were collected for transcriptomic analyses [36]. As predicted, numerous differentially expressed genes were associated with population structures and thermal stress. We also confirmed that several important pathways, associated with metabolic processes, immunity response, genetic information processing processes and digestive processes, were involved in the responses expressed between the two populations. Earlier work has already identified these pathways as critical physiological components and as involved in the responses of the aquatic animals to elevated water temperatures [37,38]. Therefore, such evidence demonstrated that gene expression variation has an interactive effect between the environmental factors and the habitat types. At the same time, the putative functional consequences of expression variation were closely related to different organisational analyses levels (transcript, gene and physiological pathway) [39]. 

### 4.1. Gene Expression Variation between Geographic Populations

Numerous differentially expressed unigenes were detected between the two studied *O. oratoria* populations, with the more upregulated unigenes obtained from the Zhoushan population (56.86% at 20 °C and 65.86% at 28 °C). Considering that two populations came from different habitat environments, it was not surprising that the majority of the gene expressed variations were observed between them. Such a phenomenon might demonstrate that adaptive differences to temperature had evolved in the ecologically divergent populations of *O. oratoria*.

Functional annotations of differentially expressed genes can provide insights about the differences of infraspecific adaptive evolution. At 20 °C, there were only two enriched GO terms that were significantly different between the two *O. oratoria* populations, which included oxidoreductase activity and acyl-CoA dehydrogenase activity. In addition, there were significant differences in some important digestive- and metabolism-related pathways between the two populations. Previous studies demonstrated that oxidative stress increases when environmental factors move away from the optimum [40,41]. Oxidative stress might reduce lipid beta-oxidation with consequent lipid accumulation, leading to hepatic steatosis [42,43]. Specifically, oxidoreductase and acyl-CoA dehydrogenase activity played important roles in lipid beta-oxidation, potentially shielding the organisms from stress effects. This process might also affect the digestive system and the metabolism differently, ultimately leading to adaptability divergences between organisms. However, the genes involved in the oxidative stress response might over-express towards a threshold when the temperature is far from optimum. Thus, many differential GO terms were observed when the two populations were exposed to 28 °C and the terms were mainly associated with cellular components. Furthermore, there were significant differences in some genetic information and immunity processes between the two populations. Most cellular activities, such as metabolic pathways, cell division and others, always occurred in the cytoplasm. The movement of Ca^2+^ in the cytoplasm represented a metabolic process signalling activity, and the ribosomes affected many cellular functions, such as repairing damage or directing chemical processes through a synthesis of proteins [44,45,46]. The significant difference in cellular component terms implied that thermal stress may cause cellular damages in the structure. The cellular stress response is a complex mechanism that aims at preventing the damage of functional proteins. Remarkably, stress responses might compromise the protein function directly through damaged cellular structures and thus present a stress signal for organisms [47,48]. In the present study, the differentially expressed genes might be associated with protein processing, antigen processing, presentation and other cellular activities and aimed at ultimately preventing cellular death. Consequently, we hypothesized that the intraspecific mutations in the same gene or different genes could lead to local adaptive divergences [49] and that the adaptability variation of different populations might correlate with habitat temperatures.

Furthermore, we also discovered that the number of differentially expressed unigenes between the populations decreased when the temperature increased (11,391 at 20 °C and 9395 at 28 °C). This trend confirmed that some functional genes of two populations might over-express towards a threshold due to the increase of the thermal stress degree [50,51,52]. On the other hand, organisms might moderate the gene expressed response over the stress degree and the gene responses of immediate survival are a priority [53]. 

### 4.2. Differentially Expressed Responses of the Two Populations Exposed to Thermal Stress

When the temperature increased from 20 °C to 28 °C, 1216 functional genes were differentially expressed and a lower proportion of up-regulated genes (20.14%) were found in the Qingdao population. In addition, several GO terms and pathways were significantly enriched in the Qingdao population when the temperature increased. The most significantly enriched biological processes were associated with the state or activity of cells, and the significantly enriched pathways were associated with genetic information, immune and environmental information processing. The most interesting finding in the present study was that the mitogen-activated protein kinase (MAPK) and the unfolded protein response (UPR) played an important role in the response to heat. The UPR presented a cellular stress response related to the endoplasmic reticulum (ER) stress and aimed to restore the normal function of the cell, degrade misfolded proteins and activate the signalling pathways associated with protein folding [47]. As a protective response, UPR might restore cell homeostasis by preventing *ER* overload with misfolded or damaged proteins. However, Han et al. considered that the functional protein synthesis and adenosine triphosphate (ATP) depletion will increase with the enhancement of stress [54]. In that case, UPR is unable to relieve the ER overload and ultimately leads to cell death [48]. On the other hand, MAPK enzymes phosphorylated and then activated the mitogen-activated protein kinase kinase 1 (MKK1) and the mitogen-activated protein kinase kinase 2 (MKK2). The latter’s phosphorylation was important for cellular proliferation, cellular cycle progression, cellular division and differentiation [55]. In addition, some MAPK enzymes might activate both the p38 and c-Jun N-terminal kinase (JNK) pathways [56]. Both the JNK and p38 signalling pathways responded to stress stimuli and were involved in cellular apoptosis or cellular differentiation. Therefore, such results indicated that thermal stress might have a more significant influence on the Qingdao population and damaging the protein function through protein denaturation. This process also represented a stress signal for UPR and MAPK. UPR and MAPK formed a complex regulatory mechanism aimed at prevented cellular damaged or apoptosis. However, we also suspected that prolonged over-expression of UPR and MAPK might ultimately lead to cellular apoptosis with the increase of thermal stress.

Our analyses also indicated that only 351 differentially expressed unigenes were obtained in the Zhoushan population due to rising temperatures, however, the stress response genes were highly upregulated (69.23%) when exposed to heat stress. At 28 °C, the differentially regulated unigenes of the Zhoushan population were primarily involved in the cellular and transcription regulation process, and the most significant pathways were those of the metabolism and the digestive system. It should be noted that the protein kinase-C (PKC) was a critical enzyme involved in the thermal response processes of the Zhoushan population. PKC is a family of protein kinase enzymes that were involved in controlling the function of other proteins [57]. This included the functions associated with receptor desensitisation, modulating membrane structure, regulating transcription, mediating immune responses, and regulating cellular activity. However, the effects of PKC were cell-type-specific [58]. Higher temperatures might lead to metabolic and digestive depressions in *O. oratoria*. In order to ensure the survival of organisms, PCKs, as functional proteins, were activated to regulate the metabolic and digestive pathways. Ibarz et al. found that prolonged fasting also caused a depletion of glycogen and such loss of energy might also activate the regulatory mechanism of the metabolic and digestive pathways [59].

We tested how thermal stress actually affected the expressed responses of two populations of *O. oratoria*. As mentioned earlier, the adaptability variation of different populations might be population-specific. Consequently, we first identified the number of differentially expressed unigenes of each population exposed to same thermal stress, respectively. Although most differentially expressed unigenes were obtained from the Qingdao population with the temperature increase, the stress response genes were highly upregulated in the Zhoushan population. Narum and Campbell also investigated the adaptive response of the functional genes in redband trout and emphasized that a lower gene variation was observed in warmth adapted populations under conditions of thermal stress [60]. Therefore, we suspected that the Qingdao population might sustain enormous thermal stress and that it should mobilize more specific genes to resist the rising of temperatures. The functional annotations of the differentially expressed genes provided insights about potential regulatory and physiological mechanisms for mediating the adaptation to thermal stress. Despite the fact that the number of differentially expressed genes was lower than in the Qingdao population, it is important to note that there were more significantly enriched GO terms and pathways in the Zhoushan population. Such results might confirm that several functional genes with large pleiotropic effects are applied in response to thermal stress in the Zhoushan population. In addition, the results of the GO terms and pathways also indicated that the degree of organism damage in the Qingdao population may be more serious. Thus, we considered that different populations can embark on different solutions to cope with heat stressors [61,62] and that a thermal adaptation of the functional genes had evolved in the Zhoushan population. In short, the higher annual temperature of the East China Sea might have generated more thermal adaptive evolutionary responses in the Zhoushan population.

## 5. Conclusions

Overall, our analyses have confirmed that the infraspecific gene expression variations of the *O. oratoria* were closely related with specific populations and their environmental factors. The results showed a gene expressed variation in a population-specific pattern, which indicates that the local environment could lead to evolutionary changes in gene regulation, ultimately leading to adaptive divergences. Therefore, we considered that different populations might use different solutions to cope with environmental stressors. Higher temperatures apparently generated a series of stress reactions in *O. oratoria*, such as oxidative stress, metabolic disorder, and cell apoptosis. We identified and compared the potential regulatory variations between two divergent populations under the same thermal stress conditions, a few genes with large pleiotropic effects in Zhoushan population. The discrepancy of regulated degree also indicated that thermal stress had a much stronger influence on the survival of the Qingdao population. In addition, this study revealed adaptive patterns in different organization levels (from transcript to gene to physiological pathway) of the two populations, and we suggest that the regulation mechanisms of the Zhoushan population was more efficient than that of the Qingdao population under thermal stress conditions. 

Finally, the present study provided some novel insights into the discrepancy between temperature adaptive differences of two *O. oratoria* populations. The results can also serve as a foundation for further studies aiming to explain the genetic basis of local adaptation in *O. oratoria* and other crustaceans. Accordingly, future studies will need to identify the potential functional consequences of these transcriptome variations and to test how functional genes affect the distribution of crustaceans. In addition, multiple environmental factors might cause synergistic and generate different local adaption for crustaceans. From a transcriptome perspective, future research needs to characterise transcriptome variation among crustacean populations under multiple environmental stressors.

## Figures and Tables

**Table 1:** Primer sequences of the five target unigenes, or contigs, analysed using qRT-PCR.

ID	Swiss-Annotation	Primer(5′ to 3′)	Product Length
CL17736.Contig.3_All	*HSP6C*_DROME	For_ATAATGCCGACGGGAAACC	140 bp
		Rev_ATATCAACGAGGTATGACTGA	
CL3018.Contig.1_All	*H3V1*_DICDI	For_ATGGTCGTTGCTGAGTGG	185 bp
		Rev_CAACGCCACAGCAAAT	
Unigene53382_All	*ORCT*_DROME	For_ CCACAAGGTTGGGTCT	146 bp
		Rev_ TTGGCTCTAGGTGAAT	
Unigene16740_All	*Y7791*_DICDI	For_ ACCCTCCTACTGGTCTTCC	196 bp
		Rev_ TCGGTGATGTATTGAGGC	
Unigene80423_All	*CFDP2*_TRAJA	For_ ATCCGAAATAGTCGTCAC	111 bp
		Rev_ ATCGTCCTGAAGTGGC	

**Table 2:** The numbers of raw and clean RNA-seq reads from each sample.

Populations	Replicate	Condition	Raw Reads	Raw Bases	Clean Reads	Clean Bases
Qingdao	1	20 °C/24 h	91,494,516	13,724,177,400	70,228,416	10,534,262,400
Qingdao	2	20 °C/24 h	84,027,732	12,604,159,800	69,060,346	10,359,051,900
Qingdao	3	20 °C/24 h	98,810,632	14,821,594,800	71,406,246	10,710,936,900
Qingdao	1	28 °C/24h	80,275,070	12,041,260,500	69,696,038	10,454,405,700
Qingdao	2	28 °C/24h	87,961,408	13,194,211,200	73,770,504	11,065,575,600
Qingdao	3	28 °C/24h	101,108,472	15,166,270,800	68,445,158	10,266,773,700
Zhoushan	1	20 °C/24 h	88,024,914	13,203,737,100	73,164,030	10,974,604,500
Zhoushan	2	20 °C/24 h	80,841,856	12,126,278,400	68,454,208	10,268,131,200
Zhoushan	3	20 °C/24 h	90,334,502	13,550,175,300	74,637,442	11,195,616,300
Zhoushan	1	28 °C/24h	88,929,736	13,339,460,400	74,887,104	11,233,065,600
Zhoushan	2	28 °C/24h	82,744,124	12,411,618,600	62,347,700	9,352,155,000
Zhoushan	3	28 °C/24h	76,745,954	11,511,893,100	66,281,820	9,942,273,000

**Table 3:** Results of the enrichment analysis of Gene Ontology (GO) terms of the differentially expressed unigenes between two populations (ZS_control_: Zhoushan population at 20 °C; ZS_treatment_: Zhoushan population at 28 °C; QD_control_: Qingdao population at 20 ℃; QD_treatment_: Qingdao population at 28 °C).

GO ID	Description	Cluster Frequency	Genome Frequency	*p*-Value
QD_control_-VS-ZS_control_ (20 °C)				
GO:0016491	oxidoreductase activity	269 out of 1679 genes	1055 out of 8386 genes	0.00146
GO:0003995	acyl-CoA dehydrogenase activity	13 out of 1679 genes	22 out of 8386 genes	0.04377
QD_treatment_-VS-ZS_treatment_ (28 °C)				
GO:0005737	cytoplasm	798 out of 1635 genes	3039 out of 8684 genes	1.74 × 10^−36^
GO:0044444	cytoplasmic part	638 out of 1635 genes	2357 out of 8684 genes	1.6 × 10^−30^
GO:0044391	ribosomal subunit	195 out of 1635 genes	520 out of 8684 genes	5.27 × 10^−22^
GO:0005840	ribosome	253 out of 1635 genes	748 out of 8684 genes	1.17 × 10^−23^
GO:0022626	cytosolic ribosome	157 out of 1635 genes	404 out of 8684 genes	5.44 × 10^−19^
GO:0003735	structural constituent of ribosome	233 out of 1585 genes	695 out of 8386 genes	9.98 × 10^−19^
GO:0043228	non-membrane-bounded organelle	416 out of 1635 genes	1487 out of 8684 genes	1.36 × 10^−20^
GO:0043232	intracellular non-membrane-bounded organelle	416 out of 1635 genes	1487 out of 8684 genes	1.36 × 10^−20^
GO:0005198	structural molecule activity	268 out of 1585 genes	847 out of 8386 genes	6.25 × 10^−18^
GO:0044445	cytosolic part	161 out of 1635 genes	431 out of 8684 genes	1.78 × 10^−19^

**Table 4:** Top 10 results of the enrichment analysis of GO terms of differentially expressed unigenes of each population exposed to thermostress (ZS_control_: Zhoushan population at 20 °C; ZS_treatment_: Zhoushan population at 28 °C; QD_control_: Qingdao population at 20 °C; QD_treatment_: Qingdao population at 28 °C).

GO ID	Description	Cluster Frequency	Genome Frequency	*p*-Value
ZS_control_-VS-ZS_treatment_				
GO:0004698	calcium-dependent protein kinase C activity	5 out of 49 genes	8 out of 8386 genes	4.05 × 10^−8^
GO:0019992	diacylglycerol binding	5 out of 49 genes	8 out of 8386 genes	4.05 × 10^−8^
GO:0009931	calcium-dependent protein serine/threonine kinase activity	5 out of 49 genes	10 out of 8386 genes	1.81 × 10^−9^
GO:0010857	calcium-dependent protein kinase activity	5 out of 49 genes	10 out of 8386 genes	1.81 × 10^−9^
GO:0004697	protein kinase C activity	5 out of 49 genes	11 out of 8386 genes	3.29 × 10^−7^
GO:0034389	lipid particle organisation	5 out of 54 genes	11 out of 8179 genes	1.94 × 10^−8^
GO:0007252	I-kappaB phosphorylation	5 out of 54 genes	12 out of 8179 genes	3.29 × 10^−6^
GO:0003824	catalytic activity	46 out of 49 genes	4939 out of 8386 genes	4.59 × 10^−6^
GO:0009950	dorsal/ventral axis specification	5 out of 54 genes	13 out of 8179 genes	5.33 × 10^−6^
GO:0002793	positive regulation of peptide secretion	5 out of 54 genes	14 out of 8179 genes	8.24 × 10^−6^
QD_control_-VS-QD_treatment_				
GO:0031435	mitogen-activated protein kinase kinase binding	4 out of 75 genes	5 out of 8386 genes	6.52 × 10^−6^
GO:0030968	endoplasmic reticulum unfolded protein response	5 out of 88 genes	11 out of 8179 genes	5.74 × 10^−5^
GO:0034620	cellular response to unfolded protein	5 out of 88 genes	11 out of 8179 genes	5.74 × 10^−5^
GO:0035967	cellular response to topologically incorrect protein	5 out of 88 genes	11 out of 8179 genes	5.74 × 10^−5^
GO:0004721	phosphoprotein phosphatase activity	9 out of 75 genes	109 out of 8386 genes	1.04 × 10^−6^
GO:0016020	membrane	92 out of 115 genes	5303 out of 8684 genes	0.00046
GO:0006986	response to unfolded protein	5 out of 88 genes	20 out of 8179 genes	0.00065
GO:0035966	response to topologically incorrect protein	5 out of 88 genes	20 out of 8179 genes	0.00065
GO:0016791	phosphatase activity	9 out of 75 genes	158 out of 8386 genes	0.00084
GO:0042578	phosphoric ester hydrolase activity	9 out of 75 genes	196 out of 8386 genes	0.00470

## Supplementary Materials

The following are available online at https://www.mdpi.com/2076-2615/9/7/399/s1, File S1: Sequence alignment result of COI in 12 individuals, File S2: Sequence alignment result of control region in 12 individuals.

## References

[1] Bopp L., Resplandy L., Orr J.C., Doney S.C., Dunne J.P., Gehlen M., Halloran P., Heinze C., Ilyina T., Séférian R. (2013). Multiple stressors of ocean ecosystems in the 21st century: Projections with CMIP5 models. Biogeosciences.

[2] Dissanayake A., Ishimatsu A. (2011). Synergistic effects of elevated CO_2_ and temperature on the metabolic scope and activity in a shallow-water coastal decapod (Metapenaeus joyneri; Crustacea: Penaeidae). ICES J. Mar. Sci..

[3] Sunday J.M., Bates A.E., Dulvy N.K. (2012). Thermal tolerance and the global redistribution of animals. Nat. Clim. Chang..

[4] Johansen J.L., Messmer V., Coker D.J., Hoey A.S., Pratchett M.S. (2014). Increasing ocean temperatures reduce activity patterns of a large commercially important coral reef fish. Glob. Chang. Biol..

[5] Pratchett M.S. (2005). Dietary overlap among coral-feeding butterflyfishes (Chaetodontidae) at Lizard Island, northern Great Barrier Reef. Mar. Biol..

[6] Tewksbury J.J., Huey R.B., Deutsch C.A. (2008). Putting heat on tropical animals. Science.

[7] Matos-Maraví P.F., Peña C., Willmott K.R., Freitas A.V.L., Wahlberg N. (2013). Systematics and evolutionary history of butterflies in the “*Taygetis clade*” (Nymphalidae: Satyrinae: Euptychiina): Towards a better understanding of Neotropical biogeography. Mol. Phylogenet. Evol..

[8] Yannic G., Pellissier L., Ortego J., Lecomte N., Couturier S., Cuyler C., Dussault C., Hundertmark K.J., Irvine R.J., Jenkins D.A. (2014). Genetic diversity in caribou linked to past and future climate change. Nat. Clim. Chang..

[9] Lemaire P., Bernard E., Martinez-Paz J., Chim L. (2002). Combined effect of temperature and salinity on osmoregulation of juvenile and subadult *Penaeus stylirostris*. Aquaculture.

[10] Tiyagarajan V., Harder T., Qian P.Y. (2003). Combined effects of temperature and salinity on larval development and attachment of the subtidal barnacle *Balanus trigonus* Darwin. J. Exp. Mar. Biol. Ecol..

[11] Larson W.A., Seeb L.W., Everett M.V., Waples R.K., Templin W.D., Seeb J.E. (2014). Genotyping by sequencing resolves shallow population structure to inform conservation of Chinook salmon (*Oncorhynchus tshawytscha*). Evol. Appl..

[12] Zhang B.D., Xue D.X., Wang J., Li Y.L., Liu B.J., Liu J.X. (2015). Development and preliminary evaluation of a genomewide single nucleotide polymorphisms resource generated by RAD-seq for the small yellow croaker (*Larimichthys polyactis*). Mol. Ecol. Resour..

[13] Xu Q.H., Cai C., Hu X.X., Liu Y., Guo Y.N., Hu P., Chen Z.Z., Peng S.H., Zhang D.S., Jiang S.W. (2015). Evolutionary suppression of erythropoiesis via the modulation of TGF-β signalling in an Antarctic icefish. Mol. Ecol..

[14] Schroder K., Irvine K.M., Taylor M.S., Bokil N.J., Cao K.A.L., Masterman K.A., Labzin L.I., Semple C.A., Kapetanovic R., Fairbairn L. (2012). Conservation and divergence in Toll-like receptor 4-regulated gene expression in primary human versus mouse macrophages. Proc. Natl. Acad. Sci. USA.

[15] Tirosh I., Weinberger A., Carmi M., Barkai N. (2006). A genetic signature of interspecies variations in gene expression. Nat. Genet..

[16] Cheviron Z.A., Whitehead A., Brumfield R.T. (2010). Transcriptomic variation and plasticity in rufous-collared sparrows (*Zonotrichia capensis*) along an altitudinal gradient. Mol. Ecol..

[17] Morris M.R.J., Richard R., Leder E.H., Barrett R.D.H., Aubin-Horth N., Rogers S. (2014). Gene expression plasticity evolves in response to colonization of freshwater lakes in threespine stickleback. Mol. Ecol..

[18] Guo J., Liu R., Huang L., Zheng X.M., Liu P.L., Du Y.S., Cai Z., Zhou L., Wei X.H., Zhang F.M. (2016). Widespread and adaptive alterations in genome-wide gene expression associated with ecological divergence of two *Oryza* species. Mol. Biol. Evol..

[19] Wang Z., Gerstein M.M., Snyder M. (2009). RNA-seq: A revolutionary tool for transcriptomics. Nat. Rev. Genet..

[20] Humphreys D.T., Westman B.J., Martin D.I.K., Preiss T. (2005). MicroRNAs control translation initiation by inhibiting eukaryotic initiation factor 4E/cap and poly(A) tail function. Proc. Natl. Acad. Sci. USA.

[21] Marshall W.S., Grosell M. (2006). Ion transport, osmoregulation and acid-base balance. Physiol. Fishes.

[22] Cheng J., Sha Z.L. (2017). Cryptic diversity in the Japanese mantis shrimp *Oratosquilla oratoria* (Crustacea: Squillidae): Allopatric diversification, secondary contact and hybridization. Sci. Rep..

[23] Du X.W., Cai S.S., Yu C.G., Jiang X.Q., Lin L.S., Gao T.X., Han Z.Q. (2016). Population genetic structure of mantis shrimps *Oratosquilla oratoria*: Testing the barrier effect of the Yangtze River Outflow. Biochem. Syst. Ecol..

[24] Johnson D., Boyer T. (2015). East Asian Seas Regional Climatology (Version 2). National Centers for Environmental Information, NOAA. https://www.nodc.noaa.gov/OC5/regional_climate/EASclimatology/.

[25] Podrabsky J.E., Somero G.N. (2004). Changes in gene expression associated with acclimation to constant temperatures and fluctuating daily temperatures in an annual killifish *Austrofundulus limnaeus*. J. Exp. Biol..

[26] Folmer O., Black M., Hoeh W., Lutz R., Vrijenhoek R. (1994). DNA primers for amplification of mitochondrial cytochrome c oxidase subunit I form diverse metazoan invertebrates. Mol. Mar. Biol. Biotechnol..

[27] Lui K.K.Y., Leung P.T.Y., Ng W.C., Leung K.M.Y. (2010). Genetic variation of *Oratosquilla oratoria* (Crustacea: Stomatopoda) across Hong Kong waters elucidated by mitochondrial DNA control region sequences. J. Mar. Biol. Assoc. UK.

[28] Grabherr M.G., Haas B.J., Yassour M., Levin J.Z., Thompson D.A., Amit I., Adiconis X., Fan L., Raychowdhury R., Zeng Q. (2011). Full-length transcriptome assembly from RNA-Seq data without a reference genome. Nat. Biotechnol..

[29] Li H., Durbin R. (2009). Fast and accurate short read alignment with Burrows-Wheeler transform. Bioinformatics.

[30] Langmead B., Salzberg S.L. (2012). Fast gapped-read alignment with Bowtie 2. Nat. Methods.

[31] Feng D., Li Q., Yu H., Zhao X.L., Kong L.F. (2015). Comparative transcriptome analysis of the Pacific oyster *Crassostrea gigas* characterized by shell colors: Identification of genetic bases potentially involved in pigmentation. PLoS ONE.

[32] Smith N.G.C., Eyre-Walker A. (2002). Adaptive protein evolution in *Drosophila*. Nature.

[33] Ma X., Dai W., Kang J., Yang L., He S. (2016). Comprehensive transcriptome analysis of six catfish species from an altitude gradient reveals adaptive evolution in Tibetan fishes. G3 Genes Genomes Genet..

[34] Wang Y., Yang L., Zhou K., Zhang Y., Song Z., He S. (2015). Evidence for adaptation to the Tibetan Plateau inferred from Tibetan loach transcriptomes. Genome Biol. Evol..

[35] Backström N., Zhang Q., Edwards S.V. (2013). Evidence from a house finch (*Haemorhous mexicanus*) spleen transcriptome for adaptive evolution and biased gene conversion in passerine birds. Mol. Biol. Evol..

[36] Logan C.A., Buckley B.A. (2015). Transcriptomic responses to environmental temperature in eurythermal and stenothermal fishes. J. Exp. Biol..

[37] Rodnick K.J., Gamperl A.K., Lizars K.R., Bennett M.T., Rausch R.N., Keeley E.R. (2004). Thermal tolerance and metabolic physiology among redband trout populations in south-eastern Oregon. J. Fish Biol..

[38] Eliason E.J., Clark T.D., Hague M.J., Hanson L.M., Gallagher Z.S., Jeffries K.M., Gale M.K., Patterson D.A., Hinch S.G., Farrell A.P. (2011). Differences in thermal tolerance among sockeye salmon populations. Science.

[39] Passow C.N., Brown A.P., Arias-Rodriquez L., Yee M., Sockell A., Schartl M., Warren W.C., Bustamante C., Kelley J.L., Tobler M. (2017). Complexities of gene expression patterns in natural populations of an extremophile fish (*Poecilia mexicana*, Poeciliidae). Mol. Ecol..

[40] Pörtner H.O. (2002). Climate variations and the physiological basis of temperature dependent biogeography: Systemic to molecular hierarchy of thermal tolerance in animals. Comp. Biochem. Phys. A.

[41] Pörtner H.O., Peck L., Somero G. (2007). Thermal limits and adaptation in marine Antarctic ectotherms: An integrative view. Philos. Trans. R. Soc. A.

[42] Sala-Rabanal M., Sánchez J., Ibarz A., Fernández-Borràs J., Blasco J., Gallardo M.A. (2004). Effects of low temperatures and fasting on hematology and plasma composition of Gilthead sea bream (*Sparus aurata*). Fish Physiol. Biochem..

[43] Leaver M.J., Boukouvala E., Antonopoulou E., Diez A., Favre-Krey L., Ezaz M.T., Bautista J.M., Tocher D.R., Krey G. (2005). Three peroxisome proliferator-activated receptor isotypes from each of two species of marine fish. Endocrinology.

[44] Savir Y., Tlusty T. (2013). The ribosome as an optimal decoder: A lesson in molecular recognition. Cell.

[45] Brandman O., Stewart-Ornstein J., Wong D., Larson A., Williams C.C., Li G., Zhou S., King D., Shen P.S., Weibezahn J. (2012). A ribosome-bound quality control complex triggers degradation of nascent peptides and signals translation stress. Cell.

[46] Shen P.S., Park J., Qin Y., Li X., Parsawar K., Larson M.H., Cox J., Cheng Y.F., Lambowitz A.M., Weissman J.S. (2015). Rqc2p and 60S ribosomal subunits mediate mRNA-independent elongation of nascent chains.elongation of nascent chains. Science.

[47] Ron D., Walter P. (2007). Signal integration in the endoplasmic reticulum unfolded protein response. Nat. Rev. Mol. Cell Biol..

[48] Imrie D., Sadler K.C. (2012). Stress management: How the unfolded protein response impacts fatty liver disease. J. Hepatol..

[49] Hoekstra H.E., Hirschmann R.J., Bundey R.A., Insel P.A., Crossland J.P. (2006). A single amino acid mutation contributes to adaptive beach mouse color pattern. Science.

[50] Mininni A.N., Milan M., Ferraresso S., Petochi T., Di M.P., Marino G., Livi S., Romualdi C., Barqelloni L., Patarnello T. (2014). Liver transcriptome analysis in gilthead sea bream upon exposure to low temperature. BMC Genom..

[51] Cassinelli J.D., Moffitt C.M. (2010). Comparison of growth and stress in resident redband trout held in laboratory simulations of montane and desert summer temperature cycles. Trans. Am. Fish. Soc..

[52] Narum S.R., Campbell N.R., Meyer K.A., Miller M.R., Hardy R.W. (2013). Thermal adaptation and acclimation of ectotherms from differing aquatic climates. Mol. Ecol..

[53] Somero G.N., Barnes B., Gordon M., Sato K., Hoppeler H. (2010). The physiology of climate change: How potentials for acclimatization and genetic adaptation will determine ‘winners’ and ‘losers’. J. Exp. Biol..

[54] Han J., Back S.H., Hur J., Lin Y.H., Gildersleeve R., Shan J.X., Yuan C.L., Krokowski D., Wang S.Y., Hatzoglou M. (2013). ER-stressinduced transcriptional regulation increases protein synthesis leading to cell death. Nat. Cell Biol..

[55] Pearson G., Robinson F., Beers G.T., Xu B.E., Karandikar M., Berman K., Cobb M.H. (2001). Mitogen-activated protein (MAP) kinase pathways: Regulation and physiological functions. Endocr. Rev..

[56] Cargnello M., Roux P.P. (2011). Activation and function of the MAPKs and their substrates, the MAPK-activated protein kinases. Microbiol. Mol. Biol. R..

[57] Wilson C.H., Ali E.S., Scrimgeour A.N., Martin A.M., Hua J., Tallis G.A., Rychkov G.Y., Barritt G.J. (2015). Steatosis inhibits liver cell store-operated Ca^2+^ entry and reduces ER Ca2+ through a protein kinase C-dependent mechanism. Biochem. J..

[58] Ali E.S., Hua J., Wilson C.H., Tallis G.A., Zhou F.H., Rychkov G.Y., Barritt G.J. (2016). The glucagon-like peptide-1 analogue exendin-4 reverses impaired intracellular Ca^2+^ signalling in steatotic hepatocytes. BBA-Mol. Cell Res..

[59] Ibarz A., Blasco J., Gallardo M.A., Fernández-Borràs J. (2010). Energy reserves and metabolic status affect the acclimation of gilthead sea bream (*Sparus aurata*) to cold. Comp. Biochem. Phys. A.

[60] Narum S.R., Campbell N.R. (2015). Transcriptomic response to heat stress among ecologically divergent populations of redband trout. BMC Genom..

[61] Koolhaas J.M. (2008). Coping style and immunity in animals: Making sense of individual variation. Brain Behav. Immun..

[62] Schulte P.M. (2014). What is environmental stress? Insights from fish living in variable environments. J. Exp. Biol..

